# Efficacy and safety of warm needle acupuncture in knee osteoarthritis: A protocol for systematic review and meta-analysis

**DOI:** 10.1097/MD.0000000000023596

**Published:** 2020-12-11

**Authors:** Ying Wei, Nairong Yuan, Jiru Ding, Lixia Wang, Yan Dong, Lu Deng, Qi Yang

**Affiliations:** a Hospital of Chengdu University of Traditional Chinese Medicine, Chengdu, Sichuan Province; b Shanxi Provincial People's Hospital, Taiyuan, Shanxi Province, China.

**Keywords:** knee osteoarthritis, protocol, systematic review and meta-analysis, warm needle acupuncture

## Abstract

**Background:**

Knee osteoarthritis (KOA) is a chronic disease, which is also recognized as a common disease affecting the elderly. However, the application of Western medicine is limited in clinical because of its obvious adverse reactions. Warm needle acupuncture (WNA) has a long history in the treatment of KOA and is widely used in Chinese. Here we will submit a protocol to evaluate the efficacy and safety of WNA in the treatment of KOA.

**Methods:**

We will search 5 English databases (PubMed, MEDLINE, Embase, Cochrane Library, Web of Science), 4 Chinese databases [China National Knowledge Infrastructure (CNKI), China Biology Medicine, Chinese Science and Technology Journal Database (VIP), and Wanfang database] and *grey literature* for randomized controlled trials of WNA in the treatment of KOA. The primary outcome measure is Western Ontario and McMaster Universities Arthritis Index (WOMAC), and *the secondary outcome will include degree of knee flexion and adverse events caused by WNA, such as dizziness, nausea, abdominal pain, arrhythmia, etc.* The selection of the literatures will be conducted by endnote X7 software, and we will use Review Manger V.5.3 software to conduct the meta-analysis.

**Results:**

This study will provide reliable evidence for WNA in the treatment of KAO.

**Conclusion:**

The conclusion of this study will testify the efficacy and safety of WNA in the treatment of KAO.

**Registration:**

OSF Preregistration. 2020, October 11; osf.io/bu5qw

## 1 Introduction

Knee osteoarthritis (KOA) is one of the most common joint disease worldwide and also a leading cause of disability and damaged quality of life.^[1]^ In addition, data from the Osteoarthritis Initiative Study indicated that osteoarthritis (OA) of lower limb may negatively impact people's mental health.^[2]^ According to the statistics, about 27 million Americans suffer from knee OA,^[3]^ and approximately 59% OA occurs in adults aged 65 years and older.^[4]^ Aside from impairing physical function and quality of life, OA also brings about a heavy economic burden, accounting for $128 billion per year in direct and indirect costs.^[5]^ More seriously, with the demographic change to a more aging population, the number of people with OA and financial expenditure also increases.^[6]^

Clinically, the clinical symptoms of KOA include joint pain, stiffness, limitation of movement, occasional articular effusion, and varying degrees of local inflammation. The main pathological feature is characterized by the loss of articular cartilage and subchondral bone sclerosis, which is caused by the synergism of mechanical mechanism and biological mechanism.^[7]^ The preliminary stage of KOA is treated conservatively, such as health education, activity modification, physical therapy, and weight loss. Anti-inflammatory and/or analgesic drugs, intra-articular hyaluronic acid and/or steroid injection, arthroscopic lavage, and debridement are used when conservative treatment fails. Total knee arthroplasty is the main treatment for moderate-to-severe pain and/or disability in the later stage of the disease.^[5]^ Though patients with KOA can receive multiple treatments, most of them suffered from obvious limitation due to the side effects.

Warm needle acupuncture (WNA) is a treatment method with the characteristics of traditional Chinese Medicine (TCM), which is a kind of therapy combining the advantages of acupuncture and moxibustion. According to theory of TCM, WNA has the efficacy of warming and dredging meridians, regulating Qi and blood circulation, and relieving pain.

With the deepening of research, it has been found that WNA can achieve the goal of treating diseases, by regulating blood circulation, nervous system, immune function, and other complex mechanisms.^[8]^ WNA is widely used to treat KOA, and its curative effect is considered equal or better than acupuncture in the clinical practice of traditional Chinese medicine. However, to the best of our knowledge, there is no critical appraisal of the evidence for WNA in the treatment of KOA. Therefore, the purpose of this review is to systematically summarize clinical researches of WNA in the treatment of KOA, and the findings of this review will be trustworthy within evidence of clinical studies.

## 2 Methods

### 2.1 Protocol registration

To ensure the authenticity and credibility of results, the research protocol was registered on the Open Science Framework. Registration: Open Science Framework Preregistration. 2020, October 11; osf.io/bu5qw, based on the items of Preferred Reporting Items for Systematic Reviews and Meta-Analyses (PRISMA).

### 2.2 Inclusion criteria of literature

#### 2.2.1 Participants of studies

We will include patients that have been diagnosed as KOA by clinicians, according to the diagnostic criteria of KOA formulated by the American Society of Rheumatology or Orthopedic Group of Chinses Medicine Association. There will be no restriction on gender, race, age, and occupation.

#### 2.2.2 Interventions of studies

In accordance with the purpose of our study, randomized controlled trials (RCTs) with WNA in the experimental group will be included. However, studies will be excluded if the intervention is a combination of WNA and other therapies. The control group was treated with conventional nonsurgical medicine therapy, such as nonsteroidal anti-inflammatory drugs, glucosamine, intra-articular injection of hyaluronic acid or steroid, and arthroscopic lavage and debridement.

#### 2.2.3 Outcome measures of studies

The primary outcome measure is Western Ontario and McMaster Universities Arthritis Index (WOMAC), which was divided into pain score, stiffness score, and daily activity score. And the total pain, stiffness, and daily activity scores were 20, 8, and 68, respectively. The secondary outcome measure will include degree of knee flexion and adverse events caused by WNA, such as dizziness, nausea, abdominal pain, arrhythmia, etc.

#### 2.2.4 Design types of studies

All available RCTs of WNA in the treatment of KOA will be included. Others such as retrospective studies, case report, reviews, and prospective studies will be excluded. The language of RCTs will be limited to Chinese and English.

### 2.3 Literature retrieval and screening

#### 2.3.1 Retrieval strategy

To obtain relevant RCTs comprehensively, we will search PubMed, MEDLINE, Embase, Cochrane Library, Web of Science, China National Knowledge Infrastructure (CNKI), China Biology Medicine, Chinese Science and Technology Journal Database (VIP), and Wanfang database systematically from their inception to 30 September 2020. The search strategies of Medical Subject Headings (MeSH) and non-MeSH terms, including “Osteoarthritis, Knee,” “Knee Osteoarthritis,” “Knee Osteoarthritides,” “Osteoarthritis of Knee,” “Osteoarthritis of the Knee,” “warm needle acupuncture,” “Warming needle,” “warm needle” and “needle warming therapy,” will be adjusted with the change of database. In addition, grey literature such as trial register, conference summary, and dissertation were searched for supplement. The language, race, or country of the participants will not be restricted. Take PubMed as an example, the detailed retrieval strategy will be:

1. Knee Osteoarthritides[Title/Abstract] OR Knee Osteoarthritis[Title/Abstract] OR Osteoarthritis of Knee[Title/Abstract] OR Osteoarthritis of the Knee[Title/Abstract] OR Osteoarthritis, Knee[Mesh]
2. Warm needle acupuncture[Title/Abstract] OR Warming needle[Title/Abstract] OR Warm needle[Title/Abstract] OR Needle warming therapy[Title/Abstract]
3. #1 AND #2

#### 2.3.2 Literature selection

**Figure 1:**
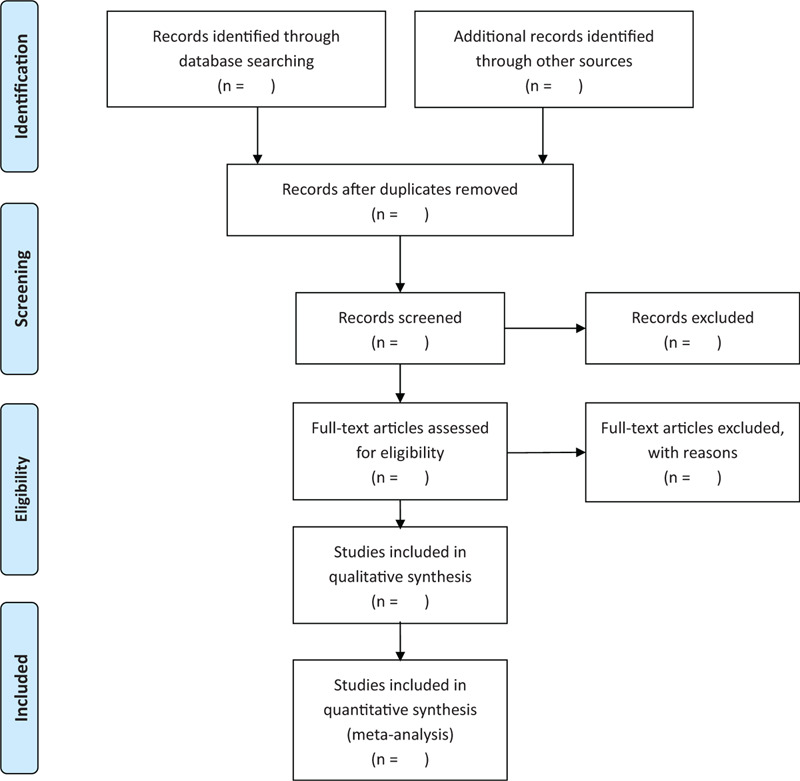
Flow chart of study selection.

All the literature retrieved from the above databases will be imported into Endnote X7, which is a literature management software. To ensure the repeatability of literature screening, 2 researchers will conduct the work independently. Firstly, studies that were obviously unqualified will be excluded after assessing the titles and abstracts of the imported articles. Then, by reading the full text in detail, the inclusion of studies will be confirmed. For the literature related to the research content but not meeting the inclusion criteria, researchers should mark the exclusion reasons separately. Any differences of opinion raised from the 2 reviewers will be solved by discussion, and the third researcher will intervene and arbitrate if they fail to achieve an agreement. Based on the items of PRISMA guidelines, the process of literature selection is presented with flow chart^[9]^ (Fig. 1).

### 2.4 Data extraction and analysis

#### 2.4.1 Data management and extraction

Data will be extracted by 2 independent researchers according to the preestablished data collection form, after reading the full text of each included literature. We will extract and register the general information, such as country, first author, year of publication, study design, basic condition of the patients, gender ratio, age, sample size and number of dropouts, follow-up time, details of intervention, outcome measures, sources of funding and adverse events caused by WNA. If incomplete data is found in the extraction process, we will contact the author and obtain the detailed data via e-mail or telephone. Any diverse opinions raised from data extraction will be solved by discussion. If necessary, the third reviewer will examine the results of data extraction and judge the unresolved disagreements.

#### 2.4.2 Dealing with incomplete data

We should contact with the corresponding author immediately to obtain relevant data via telephone or e-mail, when the data is incomplete. In another case, statistical analysis will be conducted with the existing data, and we will discuss the potential impact of missing data on the conclusion.

#### 2.4.3 Assessing the risk of bias (RoB) in included studies

Two researchers will conduct the assessment independently via using Cochrane Collaboration's risk of bias tool, which mainly involved sequence generation, allocation concealment, blinding, incomplete outcome data, selective outcome reporting, and other sources of bias. Similarly, we will resolve the disagreements arising from the evaluation process via discussion, and a third assessor will intervene and arbitrate. Generally, the RoB of high-quality RCTs has little impact on the results of the study. However, if the quality of included RCTs is uneven, the possible impact of RoB on the total effect should be analyzed, or only the results with low RoB will be combined.

#### 2.4.4 Synthesis of results

We will use Review Manger V.5.3 software (Cochrane, London, UK) to conduct meta-analysis, and the results will be presented in the form of forest maps. If the result of overall effect test is *P* < .05, it means that difference between the experimental group and control group is statistically significant. On the contrary, the difference is not statistically significant. As for the index of effect size, we will use the standardized mean difference to evaluate the continuous variables, while using rate ratio for dichotomous variables. The confidence intervals for both continuous and dichotomous variables will be set to 95%.

#### 2.4.5 Heterogeneity test

The heterogeneity among the studies will be evaluated by Higgins *I*^2^ test. And, the random effect model shall be adopted if *I*^2^ > 50%; otherwise, the fixed effect model will be used. For cases with obvious heterogeneity, subgroup analysis or sensitivity analysis will be performed to explore the source of heterogeneity and its impact on the research results.

#### 2.4.6 Evaluation of reporting bias

We will use funnel plot to detect publication bias. If the points of funnel plot are scattered and asymmetric, it will be analyzed that there is publication bias and the reliability of the results is low. In addition, Egger test can also be used to detect publication bias, and no obvious publication bias is existed if *P* > .05.

#### 2.4.7 Analysis of sensitivity

The combination of different research data is bound to produce heterogeneity. By excluding the included literature, respectively, we will analyze the changes of heterogeneity and other merger effects after excluding the corresponding literature, and infer the literature that causes heterogeneity. Then, from the design of the experiment, sample size, outcome indicators, and other aspects, we will analyze why the RCT is the source of heterogeneity. However, if the heterogeneity results are stable after excluding the literature, respectively, our research results can be considered as reliable.

#### 2.4.8 Analysis of subgroup

In the condition of obvious heterogeneity and adequate trials, we will conduct the subgroup analysis to analyze the source of heterogeneity, on the basis of difference in interventions, controls, participant characteristics, course of treatment, dose gradients, and outcome measures. For instance, included trials were divided into 2 subgroups basis of different dose gradients, and the 2 subgroups were proved to be homogeneous, respectively, via heterogeneity test. It indicated that dose factor was the source of heterogeneity if the data of the 2 subgroups were combined and detected significant heterogeneity. On the contrary, it showed that the dose has little influence on the research results.

#### 2.4.9 Ethical review and literature publication

Our study does not involve ethical review for the data of this subject are extracted from the published RCTs and have no relation with individual patients. This systematic review will conclude the efficacy and safety of WNA in the treatment of KOA, and will be published in peer-reviewed journals or conference reports.

#### 2.4.10 Assessment of evidence quality

Based on the Grading of Recommendations Assessment, Development, and Evaluation (GRADE) guidelines,^[10]^ the quality of evidence obtained in our project will be divided into 4 levels: high, medium, low, and very low.

## 3 Discussion

Osteoarthritis is a disease with multiple risk factors and complicated pathogenesis. The common risk factors include physical factors (age, female gender, obesity, family history) and local harmful mechanical factors (trauma, malalignment, and systemic relaxation).^[11]^ In clinic, knee is the joint most commonly affected by OA. KOA can lead to physical pain, joint dysfunction, poor quality of life, and huge economic burden to individuals and society. According to the statistics, about 10% to 25% of people over the age of 60 years were suffered from KOA.^[12]^ However, there is no consensus on the best therapeutic schedule to improve the symptoms of KOA at present, as the conventional western medicine used to treat KOA is accompanied by various of side effects.^[13]^

In traditional Chinese medicine, KOA belongs to the category of “arthralgia syndrome,” and the earliest written records of KOA is found in “Huangdi Neijing.”^[14]^ As one of the external therapy of TCM, WNA has the advantages of obvious efficacy, few side effects, and simple to operate in the treatment of KOA.^[15]^ With the help of heat produced by moxibustion, WNA can stimulate body's acupuncture point to achieve the function of treating and preventing diseases.^[16]^ It has been found that WNA can regulate immune response, cell proliferation, differentiation, and apoptosis through various signal transduction pathways, and regulate energy supply in vivo through oxidative phosphorylation and ATP synthesis pathway, thus relieving the clinical symptoms of KOA patients.^[17]^ However, evidence on the efficacy and safety of WNA in the treatment of KOA is insufficient. Therefore, it is necessary to conduct a systematic review and meta-analysis of the existing studies to obtain reliable evidence.

This is the first meta-analysis of WNA in the treatment of KOA. The results and conclusions of this study will provide clinicians with new ideas and programs for the treatment of KOA, and ultimately achieve the goal of reducing the physical, mental and economic burden of patients.

## Author contributions

**Conceptualization:** Yan Dong, Ying Wei.

**Data curation:** Ying Wei, Nairong Yuan.

**Investigation:** Lu Deng, Qi Yang.

**Software:** Lixia Wang, Jiru Ding.

**Supervision:** Yan Dong, Lu Deng.

**Writing – original draft:** Ying Wei.

**Writing – review & editing:** Nairong Yuan, Lixia Wang, Jiru Ding.
